# Location of oncogene-induced DNA damage sites revealed by quantitative analysis of a DNA counterstain

**DOI:** 10.1007/s00249-025-01755-x

**Published:** 2025-05-07

**Authors:** Greta Paternò, Silvia Scalisi, Gaetano Ivan Dellino, Mario Faretta, Pier Giuseppe Pelicci, Alberto Diaspro, Luca Lanzanò

**Affiliations:** 1 Department of Physics and Astronomy “Ettore Majorana”, University of Catania, Via S. Sofia 64, 95123 Catania, Italy; 2 Department of Experimental Oncology, IEO, European Institute of Oncology IRCCS, 20139 Milan, Italy; 3 Department of Oncology and Hemato-Oncology, University of Milan, 20122 Milan, Italy; 4 Nanoscopy and NIC@IIT, CHT Erzelli, Istituto Italiano di Tecnologia, Via Enrico Melen 83, Building B, 16152 Genoa, Italy; 5 DIFILAB, Department of Physics, University of Genoa, Via Dodecaneso 33, 16143 Genoa, Italy

**Keywords:** DNA counterstain, Euchromatin, Heterochromatin, Oncogene, DNA damage, Image cross-correlation spectroscopy (ICCS), DNA density

## Abstract

Oncogene activation is a key driver of cancer development, inducing aberrant cellular proliferation and DNA replication stress. This in turn, leads to DNA damage—which accumulates in specific genomic regions—contributing to genomic instability in cancer. However, the interplay between oncogene-induced DNA damage and chromatin organization is still poorly understood. In this study, we introduce a QUantitative ANalysis of DNA cOunterstains (QUANDO) to investigate the subnuclear localization of DNA damage in single-cell nuclei of U937-PR9 cells, an in vitro model of acute promyelocytic leukemia (APL). Using advanced imaging techniques, including DNA intensity analysis and colocalization by image cross-correlation spectroscopy (ICCS), we map DNA damage foci and correlate them with chromatin regions of different density. QUANDO is applied to dual-color confocal images of the DNA damage marker γ-H2AX and the DNA counterstain DAPI, allowing single-cell measurements of foci distribution within areas of low or high DNA density. We find that spontaneous DNA damage and DNA damage induced by the activation of PML-RARα oncogene predominantly localize in euchromatic regions. Conversely, when DNA damage is induced by the radiomimetic agent neocarzinostatin (NCS), the foci appear more evenly distributed in euchromatic and heterochromatic regions. These findings underscore the complex interplay between oncogene activation and chromatin organization, revealing how disruptions in DNA damage distribution can contribute to genomic instability and offering new insights for targeting DNA repair mechanisms in cancer therapies.

## Introduction

DNA damage encompasses any alteration to the chemical structure of DNA that disrupts its normal function, playing a pivotal role in the onset of genomic instability in cancer. The interplay between DNA damage and genomic instability is particularly evident when oncogenes become activated, further driving cancer development (Wei Dai 2014). Oncogenes, when mutated or overexpressed, induce abnormal cellular proliferation and replication stress, significantly increasing the burden of DNA damage (Negrini et al. 2010). As cells struggle to cope with this heightened stress, DNA damage accumulates in distinct nuclear regions, contributing to the progression of cancer. However, the exact mechanisms driving oncogene-induced genomic instability remain poorly understood (Graziano and Gonzalo 2017; Sarni and Kerem 2017). In addition, chromatin organization also plays a role in determining the spatial distribution of DNA damage within the nucleus. For instance, euchromatin and heterochromatin regions differ significantly in both transcriptional activity and accessibility, which in turn affect their exposure to DNA damage. Euchromatin is generally associated with active gene expression and is less condensed (Craig and Bickmore 1993; Estandarte et al. 2016), whereas heterochromatin is generally highly condensed and transcriptionally inactive (Comings 1978; Estandarte et al. 2016). In this context, an open question is represented by the role of local chromatin compaction in the onset of oncogene-induced DNA damage.

Advances in modern fluorescence microscopy have paved the way for studying molecular processes in intact cells (Hickey et al. 2021; Balasubramanian et al. 2023). Confocal imaging has provided valuable insights into the formation of DNA damage foci and repair processes in both fixed and live cells (Chansard et al. 2022; Heemskerk et al. 2023; Morgan et al. 2024), and markers like γ-H2AX have been instrumental in identifying DNA double-strand breaks (DSBs) and analyzing the extent and localization of DNA breaks within the nucleus (Mah et al. 2010).

In confocal microscopy, the association of a protein with specific chromatin regions is typically investigated using dual-color imaging and colocalization with well-established marks of transcription or epigenetic modifications (Pierzynska-Mach et al. 2023). A well-known marker of heterochromatin is H3K9me3 (trimethylation of histone H3 at lysine 9), associated with transcriptionally inactive regions and reinforcing the compact, repressive state of chromatin (Nicetto and Zaret 2019). A widely used marker of euchromatin is Pol2 (RNA Polymerase II), which directly participates in the transcription process and highlights regions of active gene expression (Bourdon et al. 2012). The labeling of epigenetic marks (e.g., H3K9me3) and/or transcriptional activity (Pol2) is typically performed using primary and secondary antibodies, while cell nuclei are identified using a DNA counterstain (Tarnowski et al. 1991; Bucevičius et al. 2018) such as DAPI (4',6-diamidino-2-phenylindole) or Hoechst dyes. Despite the ubiquitous use of DAPI (or alternative DNA dyes) as a nuclear counterstain, only a fraction of these studies exploits the quantitative information associated with the fluorescence intensity of the DNA dye in the cell nucleus (Vergani et al. 1992; Schmid et al. 2017; Cremer and Birk 2022; Gelléri et al. 2023, 2024).

Here, we describe a QUantitative ANalysis of DNA cOunterstains (QUANDO), as a simple methodology to analyze the subnuclear localization of DNA damage with respect to different chromatin compaction domains. The analysis requires dual-color confocal images of DNA damage foci (e.g., γ-H2AX) and a DNA counterstain.

The analysis provides, for each cell: (i) the average normalized DNA density in the DNA damage regions and (ii) the colocalization of the DNA damage regions with heterochromatin. More specifically, the colocalization is based on image cross-correlation spectroscopy (ICCS) (Comeau et al. 2008; Oneto et al. 2019; Cerutti et al. 2021), the spatial variant of fluorescence cross-correlation spectroscopy (Lanzanò 2018). An advantage of ICCS is that it does not require pre-segmentation of the image into objects (Comeau et al. 2008; Oneto et al. 2019; Cerutti et al. 2021). Recently, we have coupled ICCS with an iterative algorithm to automatically extract the value of colocalization between functional nuclear sites in hundreds of single cells (Cerutti et al. 2022; Privitera et al. 2024). As an application, we investigate the localization of the DNA damage induced by the PML-RARα oncogene. PML-RARα is a fusion protein resulting from a chromosomal translocation in acute promyelocytic leukemia (APL), which disrupts normal retinoic acid signaling and promotes aberrant cellular proliferation (de Thé et al. 1991; Villiers et al. 2023). In the context of our study, we utilize the U937-PR9 cell line, a derivative of the U937 cell line, engineered to express the PML-RARα fusion protein, upon ZnSO_4_ treatment. We have previously shown that the expression of PML-RARα in PR9 cells induces disruption of PML bodies and formation of speckles that colocalize with transcription sites, as revealed by ICCS-based analysis. This technique highlighted an increased fraction of transcription sites colocalizing with PML/PML-RARα, reflecting the abnormal occurrence of numerous PML-RARα microspeckles and the disruption of physiological PML body organization (Cerutti et al. 2022). We have also shown that colocalization between replication and transcription is higher in early S phase compared to middle and late S phase. PML-RARα oncogene activation preserves this pattern but increases colocalization in early S phase, indicating its effect on the coordination between replication and transcription (Privitera et al. 2024). We find that under conditions of spontaneous DNA damage and PML-RARα oncogene activation, γ-H2AX foci predominantly localize in euchromatic regions. In contrast, radiomimetic treatment with neocarzinostatin (NCS) induces γ-H2AX foci located in both euchromatin and heterochromatin regions.

## Materials and methods

### Cell culture and treatment

U937-PR9 cells were grown in RPMI-1640 medium (Sigma-Aldrich R7388) with addition of 10% Fetal Bovine Serum (Sigma-Aldrich F9665) and 1% Penicillin/Streptomycin (Sigma-Aldrich P4333), and were maintained at 37 °C and 5% CO_2_. To induce the expression of PML-RARα, the cells were incubated with 0.1 mM ZnSO_4_ solution and left growing for 8 h. As a positive control of DNA damage, the cells were treated with 0.05 μg/ml of neocarzinostatin (NCS) for 30 min. The cells were seeded on poly-L-lysine (Sigma-Aldrich P8920)-coated glass coverslips immediately before the experiments.

### Fluorescence labeling

Cells were fixed with 4% paraformaldehyde (w/v) for 10 min at room temperature and permeabilized with 0.5% Triton X-100 in Phosphate Buffer Saline (PBS) for 20 min. Cells were then blocked with 3% bovine serum albumin (BSA) in PBS and incubated in a wet chamber with primary antibody—opportunely diluted in blocking buffer (3% BSA, 0.5% Triton X-100 in PBS)—overnight at 4 °C. Cells were then extensively washed with washing buffer (0.2% BSA, 0.05% Triton X-100 in PBS) 3 × 15 min and incubated with secondary antibody—opportunely diluted in blocking buffer—for 1 h at room temperature, followed by the same washing procedure. Finally, cells were extensively washed with PBS, incubated with DNA dyes dilutions for 10 min and then mounted on glass slides with ProLong Diamond Antifade Mountant (Invitrogen P36961).

Primary antibodies used in this work are: anti-RNA polymerase II CTD repeat YSPTSPS (phospho S2) rabbit (Abcam ab5095) (hereinafter referred to as Pol2); anti-Histone H3 (di methyl K9) mouse (Abcam ab1220); anti-Phospho-Histone H2A.X (Ser139) rabbit (Invitrogen PA5-77995).

Secondary antibodies used in this work are: goat α-Rabbit Atto 594 (Sigma-Aldrich 77671) and goat α-Mouse Atto 594 (Sigma-Aldrich 76085). DNA dyes used in this study are DAPI (Thermo Fisher Scientific 62248).

### Image acquisition

All measurements were performed on a Leica TCS SP8 confocal laser scanning microscope, using a 1.40 NA 63X objective (HCX PL APO CS2 63/1.40 Oil Leica Microsystems). Excitation wavelengths/emission bandwidths were the following: Channel 1 (Ch1)—ATTO 594 (561/589–643) and Channel 2 (Ch2)—DAPI (405/410–483). The pinhole size was set to 0.8 Airy Units at a wavelength of 580 nm. Images were acquired with 2048 × 2048 pixels and 45 nm of pixel size.

### Data processing and analysis

The acquired images were pre-processed on Fiji (Schindelin et al. 2012) to obtain the suitable input files to run the used algorithms for image cross-correlation spectroscopy (ICCS) and DNA density analysis well explained in subsequent paragraphs.

The “Count Masks” (nuclei selection masks) were generated as follows: the images of the DNA channel (Ch2) were converted into binary images using the function ‘‘threshold’’ of ImageJ, using the “Default” threshold algorithm. The nuclei were identified and listed as objects using the ‘‘analyze particles’’ function and the images of the ‘‘Count Masks’’ were saved. Cells in mitosis were not included in the analysis.

The “intensity files” were generated subtracting the background from the intensity images of both channels (Ch1 and Ch2) using the function “Subtraction of Background” (rolling ball radius of 10 pixels). The rolling ball radius size corresponds approximately to the maximum size of heterochromatin features that we observed in our images. However, if features of larger size are present (e.g., Barr bodies with size of about 1 µm), the rolling ball radius should be adjusted accordingly.

The “binary masks of γ-H2AX” were generated excluding the background pixels from γ-H2AX channel (Ch1). The intensity threshold value was set at the same value when analyzing images acquired in the same experiment.

The count masks and intensity files were used for the ICCS analysis; these latter together with the binary masks of γ-H2AX were used for the DNA density analysis. As a control, ICCS and DNA density analyses were performed on untreated samples labeled with Pol2 and H3k9me3.

### Image cross-correlation spectroscopy (ICCS) analysis

The image cross-correlation spectroscopy (ICCS) analysis was based on a modified version of the ICCS algorithm (Oneto et al. 2019) (https://github.com/llanzano/ICCS), well described in Cerutti et al. (2022). The algorithm was performed in MATLAB (The MathWorks, Natick, Massachusetts). The advantage of the modified version of ICCS is the automatic calculation of ICCS parameters on single cells starting from multiple-input image files containing many cells. The main algorithm output is the parameter f_1_ (f_2_) values which represent the fraction of signal in channel 1 (channel 2) which is cross-correlated with the other channel. Values of this parameter range from 1 (maximum cross-correlation), to 0 (no cross-correlation), to − 1 (maximum repulsion) (Cerutti et al. 2022).

The colocalization fraction extracted by ICCS is analogous but not identical to the Pearson correlation coefficient (Cerutti et al. 2022). The main difference is that the ICCS parameter is calculated using the image cross- and auto-correlation functions. Specifically, the cross-correlation fraction f_1_ is defined as the ratio between the amplitude (estimated at zero spatial lag) of the cross-correlation function and the amplitude (extrapolated at zero spatial lag) of auto-correlation function of channel 2.

In this application, we cross-correlate the signal corresponding to DNA damage (γ-H2AX channel) with the signal corresponding to the DNA marker (DAPI channel). Thus, the value of the parameter f_1_ represents the fraction of colocalization of DNA damage with regions of high DNA density (heterochromatic regions).

### DNA density analysis

For each cell, we calculated the image of the normalized DNA intensity as:

1 $$I_{{{\mathrm{DNA}} - {\mathrm{norm}}}} \left( {x,y} \right) \, = \, I_{{{\mathrm{DNA}}}} \left( {x,y} \right) \, / \, I_{{{\mathrm{max}}}}$$

where I_DNA_(x,y) is the image of the DNA channel and I_max_ is the maximum value of I_DNA_(x,y) in the nucleus selection mask. The normalization step removes cell-to-cell variations of intensity due to different stages of the cell cycle, differences in staining efficiency and inhomogeneities across the field of view.

Then, we calculated the average value of the normalized DNA intensity in the region of interest (ROI):

2 $${\text{DNA density}}\, = \,< \,I_{{{\mathrm{DNA}} - {\mathrm{norm}}}} \left( {x,y} \right)\, > \,_{{{\mathrm{ROI}}}}$$

where the ROI is represented by the intersection between the nucleus selection mask and the region defined by “binary masks of γ-H2AX”. For each cell, the value of “DNA density” represents the average value of the normalized DNA intensity, calculated in the regions of interest. We expect high values of DNA density if the ROI overlaps with heterochromatic regions.

### Statistical analysis

Statistics analyses were performed using GraphPad Prism version 8.0.0 for Windows, GraphPad Software, San Diego, California USA, www.graphpad.com. For all the analyses, differences among groups were analyzed by one-way ANOVA followed by a Tukey’s multiple comparisons test. The values are expressed as mean ± s.d. and a *p* < 0.05 was accepted as significant. *T* test was performed on the control samples (H3k9me3 and Pol2).

## Results

### Workflow of the QUANDO method for DNA damage localization

**Fig. 1:**
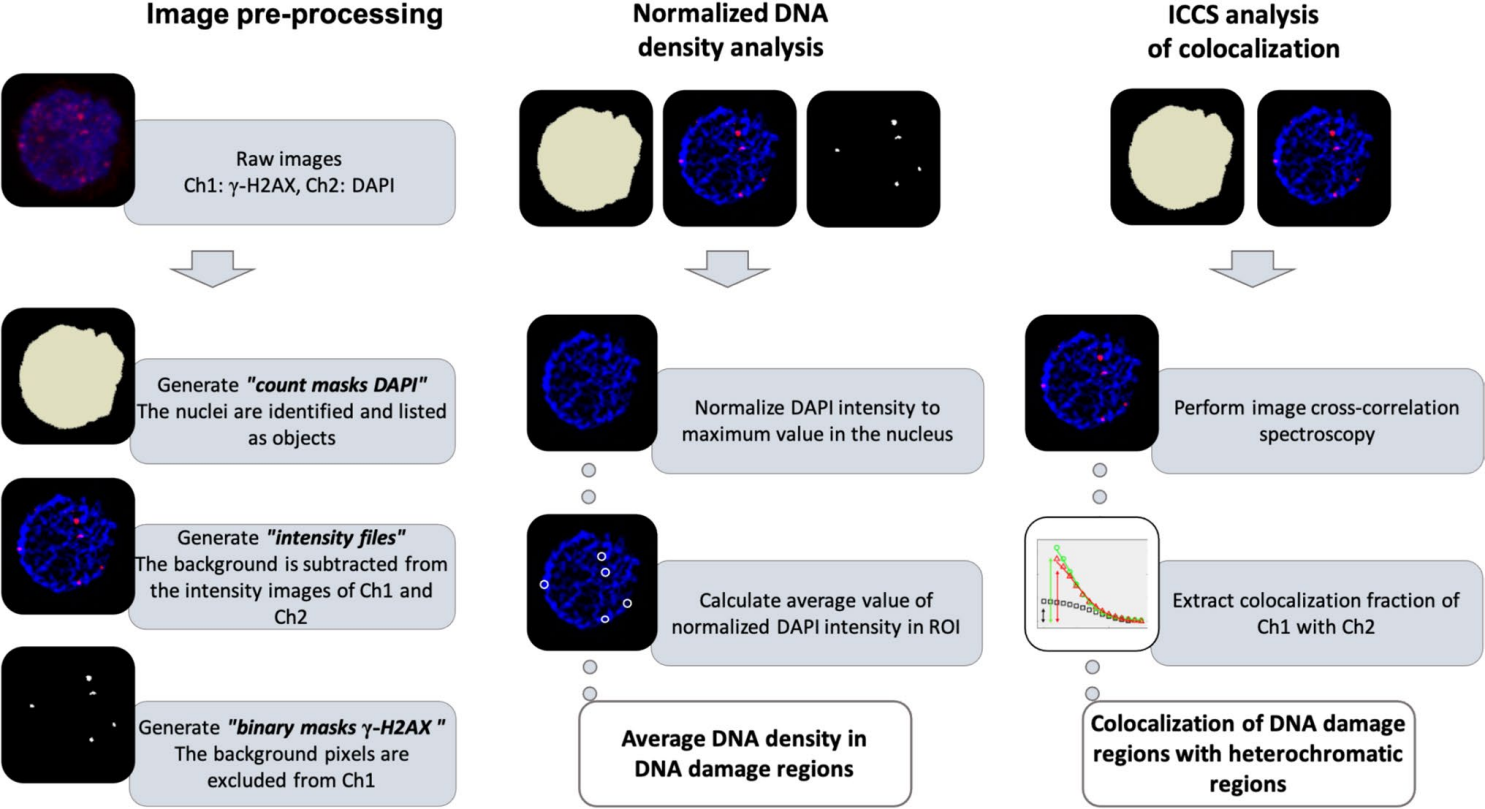
Overview of the QUANDO method. The process is divided into three phases: image pre-processing, where nuclei and DNA damage masks are generated and background is subtracted; normalized DNA density, calculated by normalizing the DAPI intensity to the maximum value in each cell and averaging it in damage regions; and ICCS analysis, which assesses the overlap between the γ-H2AX and DAPI signals to quantify the DNA damage colocalization with heterochromatin regions

The schematic workflow of the analysis is shown on Fig. 1. The raw images contain the signal corresponding to a DNA damage marker (γ-H2AX) in channel 1 (Ch1) and the signal of the DNA counterstain (DAPI) in channel 2 (Ch2). In the pre-processing step, we generate: (i) the nuclei selection masks (generated from the DAPI channel as “Count Masks” in ImageJ); (ii) the images after subtraction of background; (iii) the binary masks corresponding to the DNA damage regions (generated from the γ-H2AX channel). Note that background subtraction in the DAPI channel enhances the contrast between high-density and low-density chromatin regions. Then, for each nucleus, we evaluate the average DNA density in DNA damage regions and the colocalization of DNA damage regions with heterochromatin.

The “DNA density” is evaluated by normalizing the intensity of the DAPI channel to the maximum value in the nucleus (for an interphase cell, this maximum value corresponds to a region of heterochromatin) and by calculating its average in the region of interest (ROI) corresponding to the DNA damage foci of the same nucleus. If the ROI mainly overlaps with heterochromatic regions, we expect high values of DNA density. In contrast, if the ROI mainly overlaps with euchromatic regions, we expect low values of DNA density.

The “colocalization with heterochromatin” is evaluated by performing ICCS between Ch1 (γ-H2AX) and Ch2 (DAPI) in the selected nucleus. ICCS is a pixel-based colocalization method that does not require the segmentation of the images into objects and that has already been used for the colocalization of nuclear sites under different types of imaging modalities (Oneto et al. 2019; Cainero et al. 2021; Pelicci et al. 2022; Cerutti et al. 2022). We expect higher (lower) values of colocalization if the DNA damage regions are preferentially located in heterochromatic (euchromatic) domains of the nucleus.

### Validation of the method with markers of heterochromatin and euchromatin

As a validation, the QUANDO method was first applied to dual-color confocal images of U937-PR9 cells labeled with: (i) H3K9me3 and DAPI and (ii) Pol2 and DAPI.

**Fig. 2:**
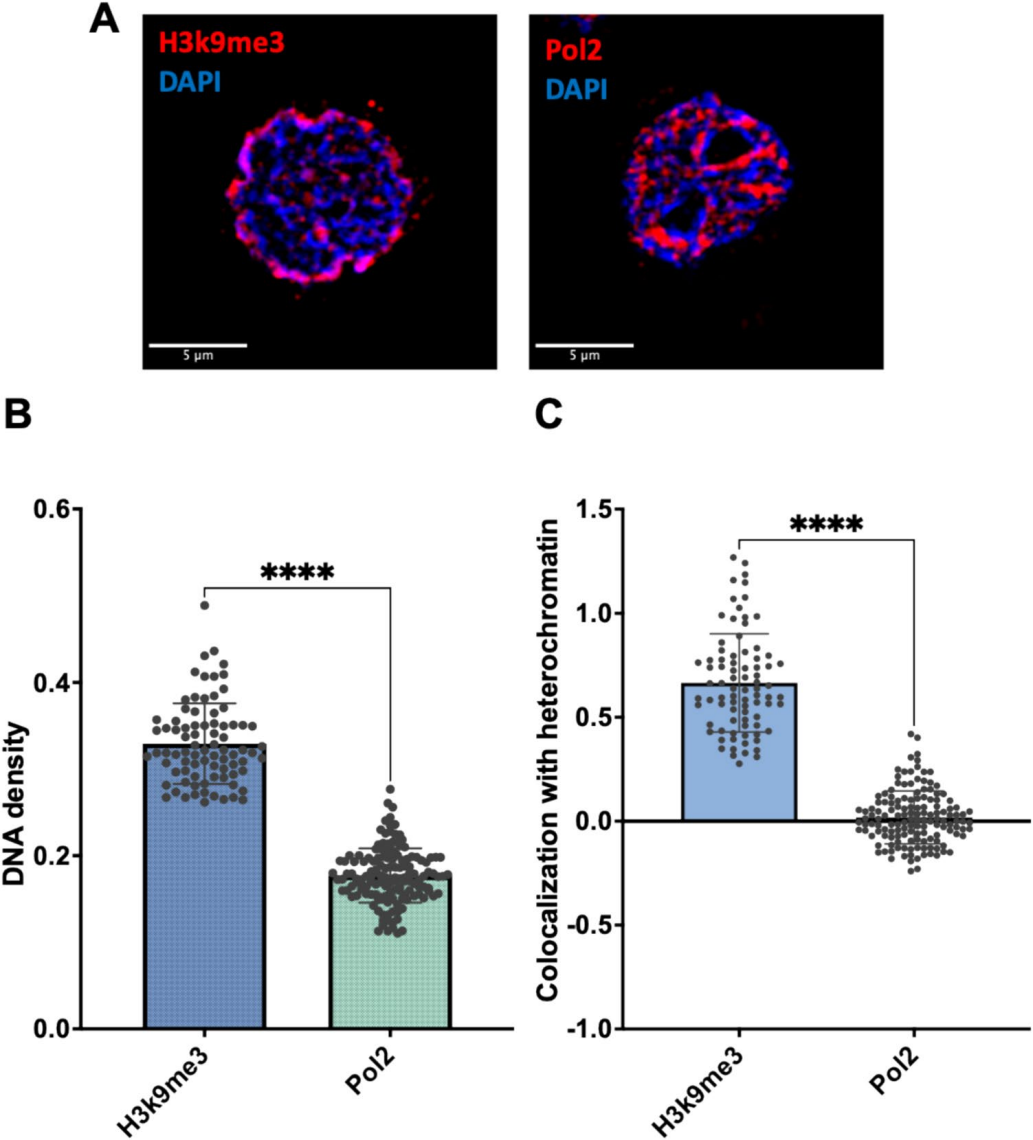
Validation of the QUANDO analysis using heterochromatin and euchromatin markers. **A** Representative confocal images of U937-PR9 cells labeled with (left) DAPI (blue) and H3K9me3 (red), or (right) DAPI (blue) and Pol2 (red). Scale bar: 5 µm. **B**, **C** Results of the QUANDO analysis: **B** DNA density corresponding to H3K9me3 and Pol2, each data point represents a single cell. *T* test ****p < 0.0001; **C** Colocalization with heterochromatin corresponding to H3K9me3 and Pol2; each data point represents a single cell, *T* Test ****p < 0.0001

H3K9me3, a histone modification linked to transcriptional repression, is an epigenetic marker specifically associated with heterochromatin, which corresponds to densely packed DNA regions that are transcriptionally silent. It plays a crucial role in maintaining chromatin structure and regulating gene expression, with its presence in high-density DAPI regions aligning with its role in stabilizing heterochromatic domains (Sánchez et al. 2019). This is visually confirmed by its prominent localization in areas of high DAPI density, which marks tightly compacted chromatin (Fig. 2A, left panel). On the other hand, Pol2 is a marker of active transcription, i.e., euchromatin, which is more loosely packed and accessible to the transcriptional machinery. Since Pol2 is directly responsible for mRNA synthesis, its presence in euchromatin strongly supports the notion that these regions are transcriptionally active and exhibit reduced chromatin compaction (Zhang et al. 2021). Thus, Pol2 predominantly localizes in low-density DAPI regions, corresponding to areas of less compact chromatin (Fig. 2A, right panel).

As expected, the DNA density corresponding to H3K9me3 was significantly higher (0.33 ± 0.05, *n* = 82 cells) than the DNA density corresponding to Pol2 (0.18 ± 0.03, *n* = 143 cells) (Fig. 2B), reflecting its preferential localization in compact, transcriptionally inactive chromatin. Similarly, the colocalization with heterochromatin was significantly higher for H3K9me3 (0.67 ± 0.24, *n* = 82 cells) compared to Pol2 (0.02 ± 0.13, *n* = 143 cells) (Fig. 2C). As shown in the box plots, H3K9me3 and Pol2 exhibit distinct clustering patterns, suggesting that the high values of DNA density and colocalization with heterochromatin obtained for H3K9me3 are consistent with its enrichment in tightly packed, transcriptionally inactive chromatin. Conversely, the low DNA density and minimal colocalization with dense DAPI regions observed for Pol2 are in keeping with its association with less compact, transcriptionally active euchromatic domains. These results provide clear evidence of the distinct chromatin environments occupied by H3K9me3 and Pol2.

These results, based on the established markers H3K9me3 and Pol2, validate the effectiveness of the QUANDO method in using the DAPI signal to extract quantitative information on the local DNA density and the proximity to heterochromatin. The strong colocalization of H3K9me3 with dense DAPI regions and the minimal colocalization of Pol2 with dense DAPI, confirmed through both ICCS and DNA density analysis, demonstrate the accuracy and reliability of this approach.

### QUANDO analysis reveals chromatin location of oncogene-induced DNA damage sites

After validating the QUANDO method on control samples, we analyzed the spatial distribution of DNA damage in U937-PR9 cells following oncogene activation. This method allowed us to analyze the relationship between the γ-H2AX and DAPI signals, assessing the chromatin-specific localization of DNA damage. γ-H2AX, a well-established marker for DNA DSBs, was employed to identify sites of DNA damage (Collins et al. 2020). Indeed, upon exposure to DNA-damaging agents, γ-H2AX accumulates at the site of DSBs, forming visible foci that can be detected using techniques such as confocal microscopy (Prabhu et al. 2024).

We first assessed DNA damage in U937-PR9 cells by quantifying the number of γ-H2AX foci under different treatment conditions. As expected, cells treated with ZnSO₄ for 8 h to activate the PML-RARα oncogene (Zn 8 h) showed a significant increase in DNA damage foci compared to untreated controls (CTRL) (mean number of foci per cell: 2.67 vs. 1.07). This increase indicates heightened genomic instability following oncogene activation. Furthermore, cells treated with NCS, a potent inducer of DNA DSBs, exhibited an even higher number of foci, confirming its effectiveness as a positive control for DNA damage (mean foci: 4.84) (data not shown).

**Fig. 3:**
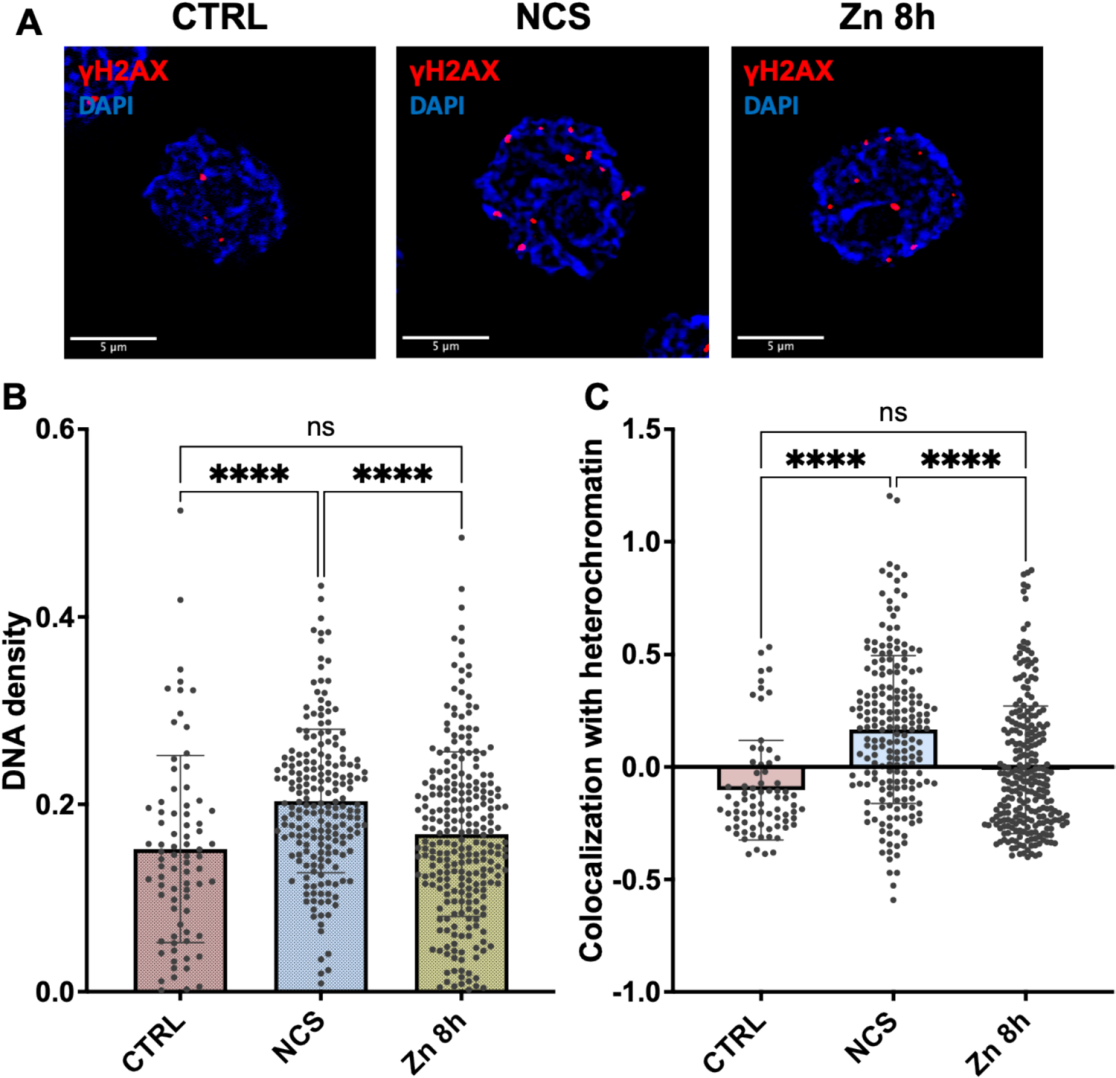
Spatial distribution of DNA damage through γ-H2AX colocalization with chromatin density. **A** Representative confocal images of U937-PR9 cells: untreated (CTRL), treated with neocarzinostatin (NCS), and treated with ZnSO₄ for 8 h (Zn 8 h). Cells are labeled with DAPI (blue) and γ-H2AX (red). Scale bar: 5 µm. **B-C** Results of the QUANDO analysis: **B** DNA density corresponding to γ-H2AX foci. Each data point represents a single cell. One-way ANOVA with Tukey’s multiple comparison, ****p < 0.0001, ns: not significant; **C** Box plot of ICCS analysis showing colocalization of γ-H2AX foci with DAPI-dense regions. Each data point represents a single cell. One-way ANOVA with Tukey’s multiple comparison, ****p < 0.0001, *ns* not significant

Confocal images were then analyzed to visualize γ-H2AX foci across three experimental conditions. In the control group (CTRL), few spontaneous DNA damage foci were observed, primarily in euchromatic regions with low-density chromatin (Fig. 3A, left). NCS-treated cells showed a significant increase in foci, which also occured in heterochromatic regions with high DAPI staining, indicating extensive DNA damage across both active and repressed chromatin (Fig. 3A, middle). Oncogene activation led to a moderate increase in foci, higher than the control but lower than NCS-treated cells, with foci mainly localized in euchromatin, suggesting damage in transcriptionally active chromatin (Fig. 3A, right).

Therefore, the QUANDO method was used to evaluate DNA damage localization by combining ICCS and DNA density analysis to assess the colocalization of γ-H2AX foci with dense and sparse DAPI regions. DNA density analysis revealed that spontaneous DNA damage occurring in control cells was associated with low DNA density (0.15 ± 0.10, *n* = 78 cells), with γ-H2AX foci predominantly located in less compact euchromatin. ZnSO₄ treatment showed similar values of DNA density (0.17 ± 0.09, *n* = 253 cells), indicating that PML-RARα oncogene-induced DNA damage was preferentially localized in euchromatin. In contrast, DNA damage in NCS-treated cells exhibited a significant increase in the value of DNA density (0.20 ± 0.08, *n* = 200 cells), indicating that γ-H2AX foci are, on average, associated to chromatin regions of higher density, and suggesting that DNA damage may occur also in heterochromatic regions (Fig. 3B).

In a similar way, the value of colocalization with heterochromatin in control cells was minimal (– 0.10 ± 0.22, *n* = 78 cells), indicating that spontaneous DNA damage mainly occurs in euchromatin regions. ZnSO₄ treatment showed similarly low values of colocalization with heterochromatin (– 0.01 ± 0.28, *n* = 253 cells), resembling the control pattern. In contrast, NCS-treated cells exhibited significantly increased colocalization with heterochromatin (0.17 ± 0.33, *n* = 200 cells), suggesting DNA damage association extended to heterochromatic regions (Fig. 3C).

Thus, the QUANDO method provides valuable insights into how different types of DNA damage influence chromatin dynamics and localization of γ-H2AX foci. The differential localization of γ-H2AX foci highlights the complexity of the DNA damage response, which varies depending on the nature of the damage and the chromatin context.

## Discussion/Conclusions

A widespread DNA counterstain for fixed cells is DAPI (4',6-diamidino-2-phenylindole). Euchromatin typically exhibits lower intensity in DAPI staining (Craig and Bickmore 1993; Estandarte et al. 2016), whereas heterochromatin dense packaging leads to more intense DAPI staining, appearing as bright regions within the nucleus (Comings 1978; Estandarte et al. 2016). The contrast between euchromatin and heterochromatin is due to differences in DNA density and composition, as DAPI preferentially binds to A–T-rich regions of DNA. In the context of oncogene-induced DNA damage in U937-PR9 cells, we used ICCS analysis to study the colocalization of γ-H2AX with DAPI, revealing an association between DNA damage and specific chromatin regions. In addition, ICCS analysis has been supported by DNA density analysis, to quantify the average concentration of DNA within distinct nuclear regions. These methods provide insights into how DNA damage correlates with chromatin density and organization, helping to map the distribution of damage foci across euchromatic and heterochromatic regions.

First, we used U937-PR9 labeled with Pol2 (to mark euchromatin) and H3K9me3 (to identify heterochromatin) as control groups to validate the method. Then, we moved on our experimental samples: untreated U937-PR9 cells, cells treated with ZnSO_4_ for PML-RARα oncogene activation, and cells treated with neocarzinostatin (NCS), as a positive control for DNA damage. The QUANDO method effectively allowed us to analyze the spatial distribution of DNA damage in the three samples. Our results demonstrate that under conditions of spontaneous DNA damage and oncogene activation, γ-H2AX foci are characterized by (i) low DNA density and (ii) poor colocalization with heterochromatin. In contrast, radiomimetic treatment with NCS revealed γ-H2AX foci associated with both euchromatin and heterochromatin. The preferential association of DNA damage with chromatin regions of different density is probably related to the different origin of the damage (spontaneous vs oncogene-induced vs radiomimetic) and might have important implications in terms of different accessibility to the DNA repair machinery.

An advantage of the proposed method is that it can be readily applied to any type of dual-color image where one channel contains the structure of interest (i.e., DNA damage foci) and the other channel contains the DNA counterstain. We note that the colocalization fraction extracted by ICCS represents a measure of the overlap between spatial intensity fluctuations in the two channels. These spatial fluctuations originate from the convolution of the fluorescent objects with the microscope point spread function. Thus, the value of colocalization depends not only on the spatial distribution of the objects (in this case, DNA damage foci and heterochromatin domains) but also on the resolution of the optical setup. In the near future, it will be interesting to apply the QUANDO method to other advanced imaging modalities including live cell imaging and super-resolution microscopy. Moreover, the recently introduced colocalization by cross-correlation (CCC) tool in Fiji, particularly useful for 3D image analysis, could further enhance the precision of our measurements by eliminating the need for data migration between applications, making it especially beneficial for complex 3D image analysis (McCall 2024).

This study aims to advance our understanding of how oncogenes, such as PML-RARα, influence the localization and accumulation of DNA damage in different chromatin regions. Ultimately, the insights gained from this innovative methodology could have significant implications for cancer research, particularly in understanding the role of chromatin architecture in genomic instability and tumor progression.

## Data Availability

The data that support the findings of this study are available from the corresponding author upon reasonable request.

## Code availability

A software that implements the QUANDO analysis is available as source code (requiring MATLAB) or a standalone distribution for Windows at https://github.com/llanzano/QUANDO.

## References

[CR1] Balasubramanian H, Hobson CM, Chew T-L, Aaron JS (2023) Imagining the future of optical microscopy: everything, everywhere, all at once. Commun Biol 6:1096. 10.1038/s42003-023-05468-9 37898673 10.1038/s42003-023-05468-9 PMC10613274

[CR2] Bourdon M, Pirrello J, Cheniclet C et al (2012) Evidence for karyoplasmic homeostasis during endoreduplication and a ploidy-dependent increase in gene transcription during tomato fruit growth. Development 139:3817–3826. 10.1242/dev.084053 22991446 10.1242/dev.084053

[CR3] Bucevičius J, Lukinavičius G, Gerasimaitė R (2018) The use of Hoechst dyes for DNA staining and beyond. Chemosensors 6:18. 10.3390/chemosensors6020018

[CR4] Cainero I, Cerutti E, Faretta M et al (2021) Measuring nanoscale distances by structured illumination microscopy and image cross-correlation spectroscopy (SIM-ICCS). Sensors 21:2010. 10.3390/s21062010 33809144 10.3390/s21062010 PMC8001887

[CR5] Cerutti E, D’Amico M, Cainero I et al (2021) Evaluation of sted super-resolution image quality by image correlation spectroscopy (QuICS). Sci Rep 11:20782. 10.1038/s41598-021-00301-x 34675304 10.1038/s41598-021-00301-x PMC8531054

[CR6] Cerutti E, D’Amico M, Cainero I et al (2022) Alterations induced by the PML-RARα oncogene revealed by image cross correlation spectroscopy. Biophys J 121:4358–4367. 10.1016/j.bpj.2022.10.003 36196056 10.1016/j.bpj.2022.10.003 PMC9703036

[CR7] Chansard A, Pobega E, Caron P, Polo SE (2022) Imaging the response to DNA damage in heterochromatin domains. Front Cell Dev Biol. 10.3389/fcell.2022.920267 35721488 10.3389/fcell.2022.920267 PMC9201110

[CR8] Collins PL, Purman C, Porter SI et al (2020) DNA double-strand breaks induce H2Ax phosphorylation domains in a contact-dependent manner. Nat Commun 11:3158. 10.1038/s41467-020-16926-x 32572033 10.1038/s41467-020-16926-x PMC7308414

[CR9] Comeau JWD, Kolin DL, Wiseman PW (2008) Accurate measurements of protein interactions in cells via improved spatial image cross-correlation spectroscopy. Mol Biosyst 4:672. 10.1039/b719826d 18493666 10.1039/b719826d

[CR10] Comings DE (1978) Mechanisms of chromosome banding and implications for chromosome structure. Annu Rev Genet 12:25–46. 10.1146/annurev.ge.12.120178.000325 85431 10.1146/annurev.ge.12.120178.000325

[CR11] Craig JM, Bickmore WA (1993) Genes and genomes: chromosome bands—flavours to savour. BioEssays 15:349–354. 10.1002/bies.950150510 8343145 10.1002/bies.950150510

[CR12] Cremer C, Birk U (2022) Spatially modulated illumination microscopy: application perspectives in nuclear nanostructure analysis. Philos Trans R Soc a: Math, Phys Eng Sci. 10.1098/rsta.2021.0152 10.1098/rsta.2021.0152 35152761

[CR13] de Thé H, Lavau C, Marchio A et al (1991) The PML-RARα fusion mRNA generated by the t(15;17) translocation in acute promyelocytic leukemia encodes a functionally altered RAR. Cell 66:675–684. 10.1016/0092-8674(91)90113-D 1652369 10.1016/0092-8674(91)90113-d

[CR14] Estandarte AK, Botchway S, Lynch C et al (2016) The use of DAPI fluorescence lifetime imaging for investigating chromatin condensation in human chromosomes. Sci Rep 6:31417. 10.1038/srep31417 27526631 10.1038/srep31417 PMC4985626

[CR15] Gelléri M, Chen S-Y, Hübner B et al (2023) True-to-scale DNA-density maps correlate with major accessibility differences between active and inactive chromatin. Cell Rep 42:112567. 10.1016/j.celrep.2023.112567 37243597 10.1016/j.celrep.2023.112567

[CR16] Gelléri M, Sterr M, Strickfaden H et al (2024) Space-time dynamics of genome replication studied with super-resolved microscopy 10.18388/pb.2021_523 39016227

[CR17] Graziano S, Gonzalo S (2017) Mechanisms of oncogene-induced genomic instability. Biophys Chem 225:49–57. 10.1016/j.bpc.2016.11.008 28073589 10.1016/j.bpc.2016.11.008 PMC5526326

[CR18] Heemskerk T, van de Kamp G, Essers J et al (2023) Multi-scale cellular imaging of DNA double strand break repair. DNA Repair (Amst) 131:103570. 10.1016/j.dnarep.2023.103570 37734176 10.1016/j.dnarep.2023.103570

[CR19] Hickey SM, Ung B, Bader C et al (2021) Fluorescence microscopy: an outline of hardware, biological handling, and fluorophore considerations. Cells 11:35. 10.3390/cells11010035 35011596 10.3390/cells11010035 PMC8750338

[CR20] Lanzanò L (2018) Back to the future: fluorescence correlation spectroscopy moves back in the cuvette. Biophys J 115:427–428. 10.1016/j.bpj.2018.06.015 30089242 10.1016/j.bpj.2018.06.015 PMC6084252

[CR21] Mah L-J, El-Osta A, Karagiannis TC (2010) γH2AX: a sensitive molecular marker of DNA damage and repair. Leukemia 24:679–686. 10.1038/leu.2010.6 20130602 10.1038/leu.2010.6

[CR22] McCall AD (2024) Colocalization by cross-correlation, a new method of colocalization suited for super-resolution microscopy. BMC Bioinform 25:55. 10.1186/s12859-024-05675-z 10.1186/s12859-024-05675-z PMC10837882 38308215

[CR23] Morgan STB, Whelan DR, Rozario AM (2024) Visualizing DNA damage and repair using single molecule super resolution microscopy. Elsevier, pp 237–245 10.1016/bs.mcb.2023.02.004 38359980

[CR24] Negrini S, Gorgoulis VG, Halazonetis TD (2010) Genomic instability: an evolving hallmark of cancer. Nat Rev Mol Cell Biol 11:220–228. 10.1038/nrm2858 20177397 10.1038/nrm2858

[CR25] Nicetto D, Zaret KS (2019) Role of H3K9me3 heterochromatin in cell identity establishment and maintenance. Curr Opin Genet Dev 55:1–10. 10.1016/j.gde.2019.04.013 31103921 10.1016/j.gde.2019.04.013 PMC6759373

[CR26] Oneto M, Scipioni L, Sarmento MJ et al (2019) Nanoscale distribution of nuclear sites by super-resolved image cross-correlation spectroscopy. Biophys J 117:2054–2065. 10.1016/j.bpj.2019.10.036 31732142 10.1016/j.bpj.2019.10.036 PMC6895719

[CR27] Pelicci S, Furia L, Scanarini M et al (2022) Novel tools to measure single molecules colocalization in fluorescence nanoscopy by image cross correlation spectroscopy. Nanomaterials 12:686. 10.3390/nano12040686 35215014 10.3390/nano12040686 PMC8875509

[CR28] Pierzynska-Mach A, Cainero I, Oneto M et al (2023) Imaging-based study demonstrates how the DEK nanoscale distribution differentially correlates with epigenetic marks in a breast cancer model. Sci Rep 13:12749. 10.1038/s41598-023-38685-7 37550322 10.1038/s41598-023-38685-7 PMC10406876

[CR29] Prabhu KS, Kuttikrishnan S, Ahmad N et al (2024) H2AX: A key player in DNA damage response and a promising target for cancer therapy. Biomed Pharmacother 175:116663. 10.1016/j.biopha.2024.116663 38688170 10.1016/j.biopha.2024.116663

[CR30] Privitera AP, Scalisi S, Paternò G et al (2024) Super-resolved analysis of colocalization between replication and transcription along the cell cycle in a model of oncogene activation. Commun Biol 7:1260. 10.1038/s42003-024-06972-2 39367096 10.1038/s42003-024-06972-2 PMC11452374

[CR31] Sánchez OF, Mendonca A, Min A et al (2019) Monitoring histone methylation (H3K9me3) changes in live cells. ACS Omega 4:13250–13259. 10.1021/acsomega.9b01413 31460452 10.1021/acsomega.9b01413 PMC6705211

[CR32] Sarni D, Kerem B (2017) Oncogene-induced replication stress drives genome instability and tumorigenesis. Int J Mol Sci 18:1339. 10.3390/ijms18071339

[CR33] Schindelin J, Arganda-Carreras I, Frise E et al (2012) Fiji: an open-source platform for biological-image analysis. Nat Methods 9:676–682. 10.1038/nmeth.2019 22743772 10.1038/nmeth.2019 PMC3855844

[CR34] Schmid VJ, Cremer M, Cremer T (2017) Quantitative analyses of the 3D nuclear landscape recorded with super-resolved fluorescence microscopy. Methods 123:33–46. 10.1016/j.ymeth.2017.03.013 28323041 10.1016/j.ymeth.2017.03.013

[CR35] Tarnowski BI, Spinale FG, Nicholson JH (1991) DAPI as a useful stain for nuclear quantitation. Biotech Histochem 66:297–302 1725854

[CR36] Vergani L, Gavazzo P, Facci P et al (1992) Fluorescence cytometry of microtubules and nuclear DNA during cell-cycle and reverse-transformation. J Cell Biochem 50:201–209. 10.1002/jcb.240500210 1331127 10.1002/jcb.240500210

[CR37] Villiers W, Kelly A, He X et al (2023) Multi-omics and machine learning reveal context-specific gene regulatory activities of PML::RARA in acute promyelocytic leukemia. Nat Commun 14:724. 10.1038/s41467-023-36262-0 36759620 10.1038/s41467-023-36262-0 PMC9911410

[CR38] Wei Dai YY (2014) Genomic instability and cancer. J Carcinog Mutagen. 10.4172/2157-2518.1000165 25541596 10.4172/2157-2518.1000165 PMC4274643

[CR39] Zhang S, Übelmesser N, Josipovic N et al (2021) RNA polymerase II is required for spatial chromatin reorganization following exit from mitosis. Sci Adv. 10.1126/sciadv.abg8205 34678064 10.1126/sciadv.abg8205 PMC8535795

